# Strategies for Success With Umbilical Cord Haematopoietic Stem Cell Transplantation in Children With Malignant and Non-Malignant Disease Indications

**DOI:** 10.3389/fcell.2022.836594

**Published:** 2022-04-06

**Authors:** Rob Wynn, Ramya Nataraj, Rubiya Nadaf, Kay Poulton, Alison Logan

**Affiliations:** ^1^ Royal Manchester Children’s Hospital, Manchester, United Kingdom; ^2^ Paediatric Blood and Marrow Transplant Programme, Manchester, United Kingdom; ^3^ Transplantation Laboratory, Manchester University NHS Foundation Trust (MFT), Manchester, United Kingdom; ^4^ Manchester University NHS Foundation Trust (MFT), Manchester, United Kingdom

**Keywords:** leukaemia, bone marrow transplant, Hurler disease, cord blood, graft *versus* host disease, immunotherapy

## Abstract

Umbilical Cord blood is an intuitively attractive stem cell source, but its use has declined since it is associated with an increased procedure-related morbidity and transplant related mortality. Some of this reflects that cord blood transplants are more often HLA-mismatched compared to other unrelated donor transplants. The ability to transplant in such a setting, indeed without high rates of chronic Graft versus Host Disease (GVHD), constitutes an advantage compared to other unrelated donor cell sources and there are other advantages specifically associated with cord blood as a donor cell source. These advantages must be weighed against its disadvantage, and we have utilised cord blood preferentially as a donor cell source in certain clinical situations in paediatric medicine. In non-malignant diseases, outcomes in metabolic disease are critically dependent on age at transplant and the enzyme delivered by that transplant, and in cord blood transplantation then the time to transplant can be minimised and the engrafted recipients have higher chimerism that delivers higher enzyme levels. In malignant diseases, studies have described reduced relapse rate and better GVHD-free survival, and so we have prioritised cord as a donor cell source where the risk of relapse is highest, and the effects of higher transplant related mortality is most clearly offset by the reduced relapse rates.

## Introduction

Cord blood use is declining. Its decline as a stem cell source in alternate donor haematopoietic stem cell transplant—when there is no matched family donor—has been mirrored by the rise in haplo-identical donor transplant (Passweg et al., 2021).

The reason for this is clear—haplo-identical donor transplant is easier, with lower procedure-related morbidity and mortality than cord blood transplant. This is principally due to the different graft composition in the two clinical settings. The stem cell dose is significantly higher in haplo-identical transplants, ensuring engraftment and transfusion independence earlier than in cord blood transplant, in which the cell dose is relatively small and limiting. Additionally, in haplo-identical transplant allo-reactive donor T-cells are removed from the graft, either during *in vitro* graft manipulation, with either positive selection of CD34 stem cells or negative selection of CD3 T-cells, including specifically αβT-cells, or *in vivo* with post-transplant cyclophosphamide. In cord blood transplant then these techniques are not employed, and allo-reactive, often HLA-mismatched, T-cells are infused with the graft, with higher rates of acute graft versus host disease (GVHD).

In transplant, however, there is no such thing as a free lunch, and losing this allogeneic effect of transplant may become disadvantageous in certain clinical circumstances. The retention of T-cells that are often mismatched to the recipient in the cord blood graft is likely important in reducing graft immunologic rejection and leukaemia relapse after transplant. Additionally, family donors, including haplo-identical ones, may be carriers of genetic illnesses and constitute inferior donors to wild-type, including cord blood, donors in certain genetic diseases, including metabolic illnesses.

This review will examine the properties of cord blood transplant that generates continuing utility as a stem cell source in transplant in children. Cord could conceivably be prioritised as the source of choice to deliver the best achievable long-term outcome, especially in children with high-risk disease. The cord unit donor selection algorithm of our institution will be presented, and the practice of our institution in cord blood transplantation in malignant and non-malignant disease will be reviewed.

## Properties of Cord Blood, as a Donor Cell Source, Relevant to Paediatric Transplant

1) Reduced time to transplant

The time to transplant is critically important in many paediatric conditions. The window of opportunity for transplant success is especially small in high-risk leukaemia and in rapidly progressive neurological, metabolic conditions. Cord blood units are available for immediate shipment, and this is attractive in these conditions requiring urgent transplant and compares favourably with unrelated donor transplant, where there is obligate delay in finding the donor, and scheduling the donor medicals and stem cell collection.

Cord blood is often used in metabolic diseases, and this partly is because of this reduced time between diagnosis and recognition of transplant need, and actual transplant with allogeneic stem cell infusion. We have shown that the age at transplant clearly affects both somatic and neurological outcomes in Hurler syndrome, MPSIH (Aldenhoven et al., 2015). Age at transplant is influenced by both age at diagnosis and the interval between diagnosis and transplant. The former can be reduced by better disease awareness and, increasingly often, by newborn screening (NBS), and the latter by using umbilical cord blood, since the donor is both available and a less stringent HLA matched donor can be utilised. Krabbe Disease is a lysosomal storage illness with rapidly progressive neurological deterioration in early infancy in its severest form. It can be clearly seen that both survival and long-term patient performance are improved in patients that are diagnosed with NBS and immediately transplanted using the best available cord blood donor than transplanting older patients (Escolar et al., 2005).

In Primary Immune deficiency (PID), cord blood is acceptable as a family donor, just as a matched sibling donor. In matched unrelated donor then matched cord is considered comparable to matched adult donor. Where there is no matched donor, then there is no direct large study comparing mismatched cord to mismatched family or unrelated donor. Studies in specific diseases such as Severe Combined Immune deficiency (SCID) and Wiskott-Aldrich Syndrome (WAS) have described that cord blood has similar outcomes to other cell sources. Where there is no matched donor then haplo-identical donors—with either *in vivo* or *ex vivo* T-cell depletion—have been used. There is no direct comparison. In HLH there is registry data that outcomes are poor with mismatched cord blood donors in this disease, and many would prefer haplo-identical donors (Furtado-Silva et al., 2019). The relationship with HLA mismatch is common to other non-malignant diseases (Eapen et al., 2017). There are few direct comparative studies between cord and haplo-identical donors in immune deficiency. For any individual patient the judgments on alternate donor selection might reflection enter expertise as well as the degree of mismatch in any available cord blood units.

In haemoglobinopathy then matched sibling donor cord blood gives comparable outcomes to matched sibling donor marrow transplant (Eapen et al., 2017; Locatelli et al., 2013). The use of the directed cord blood donation clearly saves the donor the bone marrow harvest procedure.

In thalassaemia there is good survival with continuing medical care with red cell transfusion and iron chelation and transplant is usually not offered where is not a genotypically identical family donor. In exceptional circumstances such as intolerance of chelation or return to a country with unsafe blood transfusion then alternate donor transplant may be offered. Haplo-identical or cord blood transplant might be considered but cord outcomes will be affected significantly by the degree of HLA mismatch, and haplo-identical approaches might be preferred where there is no well-matched cord blood donor (Anurathapan et al., 2020).

In sickle cell disease then published results with cord blood transplant are not good enough for it to be a validated approach, and we would prefer haplo-identical transplant in this setting where there is no matched family donor, and where there is a clear disease indication for transplant. The conditioning in cord blood approaches has often been of reduced intensity, and this is likely not to predict for sustained donor cell engraftment. There is greater experience of haploidentical donors in this setting, and this is therefore our preferred cell source in this situation (Abraham et al., 2017; Gluckman et al., 2020).

2) Cord blood increases the donor pool, even for those for whom donors are difficult to find

The unrelated donor cord blood donor pool might be thought to better represent the ethnic diversity of the current population than the unrelated donor registry, since units are taken from women delivering in areas of high ethnic diversity, rather than from adults who have volunteered to donate. This is relevant to the many paediatric patients without a matched family donor and are from an ethnic background that is underrepresented on adult donor panels (Gragert et al., 2014).

3) Cord blood transplants can be more safely mismatched at HLA antigens

**FIGURE 1:**
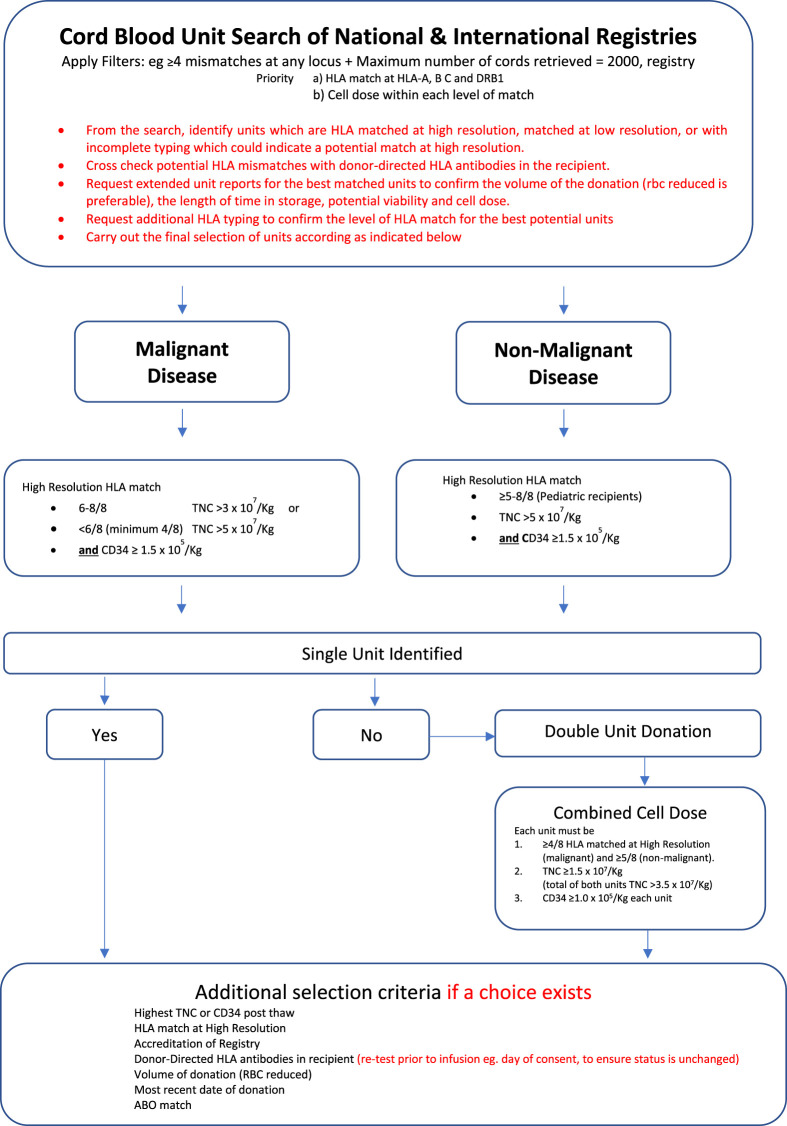
Algorithm for the selection of matched CBU donors for malignant and non-malignant (Little et al., 2021).

The utility of cord blood for those children in whom it is difficult to find fully matched family or unrelated donors is further emphasised by the better ability to utilise HLA-mismatch without compromising clinical efficacy or transplant outcomes. The algorithm used at our centre for the selection of matched cord blood unit donors is summarised in Figure 1. The algorithm covers single donor unit selection for patients with both malignant and non-malignant conditions. Figure 1 also describes an option for the selection of double cord unit donations should the only available single units matched at the required level have an insufficient cell dose. The algorithm for the selection of a double unit donation is identical for adults or paediatric recipients, regardless of their primary disease.

We contributed to the study published by Eapen et al., of 1199 paediatric cord blood transplants in children with non-malignant diseases and were reported to either the European Group for Blood and Marrow Transplant or to the Center for International Blood and Marrow Transplant Research (CIBMTR) (Eapen et al., 2017). There was no difference in transplant related mortality (TRM) or overall survival (OS) for recipients of one allele mismatched cord blood units compared to recipients of cord blood units that were fully matched at 8 alleles (class I HLA-A, -B and -C, and class II, HLA-DRB1). Both TRM and OS were increasingly adversely impacted by greater mismatches.

We compared outcomes in children with Hurler Syndrome with various graft sources in an EBMT and IBMTR study. This paper was from an earlier era when matching was less exacting and performed at antigen level at class I (HLA-A and -B only) and at high resolution only at HLA-DRB1. The transplant survival was similar between matched sibling donors and 6/6 matched cord blood unit recipients, and superior to that of 5/6 cord blood and 8/8 allele-level matched unrelated donor recipients which were similar to each other. Outcomes were least good in mismatched unrelated donor recipients (Boelens et al., 2013).

Many studies similarly report the relationship between transplant outcomes and HLA-mismatch in malignant diseases in children and adults. The interpretation of such data is more complex, since HLA-mismatch adversely impacts transplant related mortality but is reported in many series—particularly in adults to positively impact leukaemia relapse risk after transplant (Eapen et al., 2007; Eapen et al., 2014; Sanz et al., 2014; Yokoyama et al., 2020). For patients with non-malignant disorders, it is recommended not to use cords with less than a 5/6 HLA match (see Figure 1).

Eapen reported 502 children with acute leukaemia and transplanted with cord blood who were reported to the CIBMTR (Eapen et al., 2007), and compared with 283 bone-marrow recipients. Cord blood units were matched at class I (HLA-A and –B) at antigen level and at allele level for HLA-DRB1 and, notwithstanding this inferior matching, transplant outcomes were similar for cord blood units mismatched at one and two antigens compared with allele matched bone marrow and was possibly superior for fully matched (6/6) cord blood units. She extended this IBMTR survey and reported 1568 children that received CB until typed to allele level at class I (HLA-A, -B and –C) and class II, HLA-DRB1, and reported similar OS between units mismatched at 1, 2 or 3 alleles with the donor, although these were inferior to the few patients transplanted with fully matched donors (Abraham et al., 2017; Gluckman et al., 2020).

Sanz et al. have reported improved leukaemia-free survival rates in 79 adults with AML are actually significantly better in their institution when the cord blood units are mismatched to the patient (Sanz et al., 2014). Yokoyama reported outcomes on behalf of the Japan Society for Haemopoietic Cell Transplantation from 513 children and 3537 adults receiving allele-level typed single cord blood units for acute leukaemia. Significantly inferior outcomes were only in those matched at fewer than 5 alleles (<5/8 matched) (Yokoyama et al., 2020).

4) There is less disease relapse after cord blood transplant for malignant disease than after matched sibling or unrelated donor or haplo-identical donor transplant

Relapse is reduced after cord blood transplant compared to other cell sources. This has been reported in many single institution and registry studies. This does not always translate to improved OS or leukaemia-free survival since there is confounding higher TRM in those same cord blood transplant recipients.

Milano compared outcomes in Seattle of 140 cord blood transplant recipients with 344 HLA matched and 98 HLA-mismatched unrelated donors. There was reduced relapse in the cord blood group, particularly striking in the MRD positive group, and this translated to an improved OS, particularly against the mismatched unrelated donor transplant recipients (Milano et al., 2016).

In Eapen’s malignant disease IBMTR cord blood transplant survey then there was reduced relapse in the mismatched cord blood transplant recipients compared with the marrow recipients (Abraham et al., 2017; Gluckman et al., 2020). A recently reported EBMT registry study showed no significant relapse difference between adults receiving a haploidentical or single cord blood units but most of the cord blood recipients had received T-cell depleting serotherapy, which likely negatively adversely impacts relapse incidence (Giannotti et al., 2018). In the Japanese series no serotherapy was given, and Barker has reported low relapse rates in adult patients receiving double cord blood transplants, without any impact of pre-transplant MRD and in a cohort of significantly mismatched patients (Barker et al., 2020; Yokoyama et al., 2020). In a study of adult and children receiving either haplo-identical or T-replete cord blood, Wagner reported reduced relapse in the 5/8 cord recipients compared to the haploidentical or better matched cord blood transplant recipients (Abraham et al., 2017; Gluckman et al., 2020).

This likely reflects an ontogeny difference between cord blood cells, including cord blood T-cells, and adult cells, including adult T-cells. Hiwarkar demonstrated that cord blood T-cells were superior to adult T-cells in controlling disease in a xenograft human B-cell lymphoma model (Hiwarkar et al., 2015). Cord blood transplants are more likely to be mismatched than unrelated donor transplants and more likely to be performed T-cell replete than unrelated and haplo-identical transplants. It likely also reflects that the cord blood graft is more likely to be either or both T-cell replete and mismatched than in other types of transplants.

5) Engrafted cord blood transplant recipients are more likely to be fully donor engrafted than marrow donor recipients

Although the risk of primary graft rejection is increased in cord blood transplant recipients, we have reported that mixed donor chimerism is much reduced in engrafted Hurler Syndrome patients that have a cord blood transplant than a matched sibling or unrelated donor marrow or peripheral blood transplant (Lum et al., 2017). Since enzyme delivered by the graft is a product of donor engraftment and donor genotype (wild type donors delivering twice as much enzyme as family, carrier donors), then fully engrafted unrelated donors deliver more enzyme than chimeric engrafted or carrier donors. We reported that lesser enzyme delivery is associated with a poorer graft outcome, expressed as either residual substrate or residual disease manifestations (Wynn et al., 2009; Aldenhoven et al., 2015). Those patients with higher enzyme levels after transplant are more likely to grow better and receive fewer surgical interventions for disease-related carpal tunnel syndrome or cervical cord compression.

6) Even in the T-cell replete and mismatched donor transplant, the rates of chronic graft versus host disease are low

The aim of paediatric transplant is disease-free, graft versus host disease (GVHD) free survival. Chronic GVHD is particularly disabling and limits the utility of transplant in the non-malignant setting where its presence has no benefit at all to disease outcome. The rates of chronic GVHD are reduced after cord blood transplant, and even in the T-cell replete and HLA-mismatched donor setting.

Barker reported rates of cGVHD that were less than 10%, even in the double cord setting, without serotherapy and where the median match was 5/8, and none were fully matched (Barker et al., 2020). Wagner in his comparison of haplo-identical and cord blood transplants did not find any difference in rates of GVHD, even though the former is T-cell depleted and the latter are also significantly HLA-mismatched (Wagner et al., 2021).

We reported in a multi-institutional study of 317 children receiving T-cell replete single cord blood transplants for acute myeloid leukaemia that GVHD-free, leukaemia-free survival was similar in matched sibling and unrelated donor cord transplant recipients, and significantly higher than in unrelated donor recipients, even though the cord units were very significantly mismatched, and the unrelated donors were largely matched (Keating et al., 2019).

Mehta RS et al. recently reported that the incidence of GVHD-free, relapse-free survival was better in the CB group compared to 7/8 BM. In this cohort, only 16% of CB were fully matched at HLA A, B and DRB1 which shows that CB recipients can tolerate HLA mismatch better with lesser incidence of Acute and chronic GVHD compared to BM recipients (Mehta et al., 2019).

7) Cord blood transplantation has higher transplant-related mortality with acute GVHD, increased graft failure, immune cytopenia and respiratory failure

Cord blood transplantation has higher morbidity and mortality than some other transplant types. Of course, some is comparison of apples with pears. Mismatched unrelated cord blood is often compared with matched unrelated or matched family donors, and for any individual patient then no such donor was available. Milano reports a higher death rate in mismatched unrelated compared to (usually) in mismatched unrelated cord blood transplants (Milano et al., 2016), and we reported similar poor outcomes in mismatched unrelated donors compared to mismatched cord blood, and to matched donors in Hurler syndrome (Boelens et al., 2009).

Engraftment is slower with cord blood than with marrow, or haplo-identical transplant. Graft failure is the principal course of mortality after cord blood transplant for non-malignant disease (Abraham et al., 2017; Gluckman et al., 2020), and we reported that the pattern of graft failure was more likely to be primary, aplastic type in cord blood recipients compared to that seen in unrelated or sibling donor recipients, where it is more likely late autologous reconstitution (Abraham et al., 2017; Gluckman et al., 2020). Immune mediated cytopenia (IMC) is more common after cord blood and might be seen to limit the utility of its use (Deambrosis et al., 2019). Our group has associated graft failure and IMC and has reported a reduction in both after the addition of rituximab to the preparative regimen (Nataraj et al., 2022).

Intestinal failure prolongs hospital admission after cord blood transplant. This may be because of GVHD, and Barker reported that the GI tract was the organ most commonly involved in acute GVHD after such transplant (Herrera et al., 2011; Gupta and Masia, 2013). However not all intestinal failure or biopsy-proven enterocolitis is due to GVHD and specific, cord blood transplant associated “cord colitis” has been reported, although its pathogenesis, and its optimal management remain uncertain (Herrera et al., 2011; Gupta and Masia, 2013).

CB recipients have delayed immune reconstitution post HSCT. Studies suggest that there is delayed viral-specific immune reconstitution leading to higher incidence of viral reactivation in this cohort (Servais et al., 2017). This is more common when T cell depletion methods are used to prevent GVHD. In the T-cell replete setting then recovery is accelerated and there is more acute, but not chronic, GVHD and there are fewer virus infections. In the T-deplete setting then recovery is indeed delayed, just as it is in the T-deplete adult donor setting (Herrera et al., 2011; Gupta and Masia, 2013). We have noted that much of virus control after HCT is mediated by autologous, persisting, virus-specific T-cells (Sellar et al., 2015; Nadaf et al., 2021). The improved donor chimerism of the cord HCT, including lymphoid chimerism, implies that such autologous, virus-specific T-cells will be cleared after cord HCT. Failure to control virus is therefore a manifestation as much of the efficacy of the cord in clearing memory cells of the recipient, as it is of the cord delayed immune reconstitution. Whether or not CB transplant predisposes patients to higher risk of infections in the later post-transplant (>100 days) period is less defined. There was no significant difference in the infection rates beyond day 100 in both CB and BM-recipient groups in the CIBMTR registry analysis (Servais et al., 2017).

## Selection of Potential UCB Donors

Taking measures to identify a donor which would offer lifelong immunological accommodation is key to a successful programme. Selection of the optimal cord blood unit involves consideration of HLA and non-HLA factors outlined in Figure 1. Initially, high resolution HLA typing of the patient at HLA-A, B, C, DRB1, DRB3/4/5, DQA1, and DQB1. HLA-C and HLA-DQ is used to identify unconventional linkage which can be used during the search by increasing the likelihood of identifying donors with shared ancestral haplotypes.

Simultaneous national and international donor searches are initiated using targeted filters to optimize the potential number of donors for review. Useful search filters include applying the≤4 mismatches on any locus filter, setting a minimum TNC threshold and restricting choice to frequently used or United Kingdom registries. Other factors that should be considered include the accreditation status of the stem cell bank, the age of the donation, as more recently processed donors use improved methods of isolation, red cell reduction and have higher post thaw viability. A suggested upper limit of 2000 units for review should be retrieved during a search.

Potential units are shortlisted based on optimum HLA match out of 8 (HLA -A, B, C and DRB1) without a locus bias, if possible, avoiding mismatching both specificities at any one locus. Extended matching is essential for patients with non-malignant disorders. Where HLA data are missing and an unusual association between loci exists, it is helpful to use HLA haplotype searches in websites such as allelefrequencies.net (Gonzalez-Galarza 2020) (Gonzalez-Galarza et al., 2020) to select potential populations in which the patient’s unusual association is most likely to be identified. Additional HLA typing may then be selectively requested for potential donors with shared ethnicity. Units of equivalent rank are stratified using the HLA match grade, TNC/kg, CD34 enumeration, frozen volume, ABO blood group, viability, whether the unit has been red blood cell reduced, colony forming units, any specific maternal risk factors identified. In mismatched cord blood units, data on non-inherited maternal antigens can be used to select mismatched donors which will be better tolerated post-transplant. This is not an exhaustive list, and the full details of the factors are summarized in the BSHI 2021 guidelines (Little, 2021) (Little et al., 2021).

From the final shortlist of donor units, up to five cord blood units are selected and extended HLA typing requested to confirm the high-resolution match with the patient. Where a choice exists, HLA mismatches in the donor must be selected with consideration of the patient’s HLA antibody profile at the time of transplant, avoiding high titre specificities. The final selection from the shortlist will prioritize the best HLA matched unit with the largest viable cell dose.

## Manchester Children’s Donor Selection Algorithms in Malignant Disease

We prioritize cord blood in difficult-to-cure myeloid disease. We recognize that there is a higher procedure related risk using mismatched unrelated cord blood as a donor to be using matched unrelated donor. However, we further recognize that there is a reduced risk of relapse using the cord blood in a T-cell replete setting, and that the risk of chronic GVHD is low. In children therefore with relapse after a previous transplant or who are MRD positive after chemotherapy, then we will use a cord blood donor, as a platform to immunotherapy of refractory disease. In children with higher risk disease the better disease outcomes overcome the higher risk of such a transplant.

In better risk myeloid disease, then we will follow our usual donor hierarchies of matched sibling donor, before matched unrelated donor (10/10 allele level matched) or matched cord blood donor (8/8 allele level matched), and if there is no matched donor then we would consider mismatched cord blood donor or haplo-identical donor, before mismatched unrelated donor.

In ALL we use the same donor hierarchy but in B-lineage ALL that is refractory (MRD >10^−4^) we use chimeric antigen receptor (CAR) T-cell therapy, and we consider refractory T or bilineage, including infant leukaemias, as similar to refractory myeloid malignancy.

## Manchester Children’s Donor Selection Algorithms in Non-Malignant Disease

In general, our donor hierarchy in non-malignant disease is the same as in good-risk malignant disease or in ALL. We will use a matched donor (including a matched (8/8) cord blood donor) before a mismatched donor. For a family donor, we would consider a directed cord blood donation from a matched sibling to be equivalent to the bone marrow donation, if the cell count is satisfactory. We will use a mismatched cord blood donor in preference to a mismatched unrelated donor, and in all such diseases the conditioning regimen includes T-cell depleting serotherapy.

We will prefer to use cord blood where there is urgency to get to transplant such as in neurological, metabolic diseases and where full donor chimerism is associated with a superior clinical outcome, including metabolic diseases.

In other situations, in non-malignant diseases, then judgment is required in selection of alternate donors. Where there is disease that has acceptable non-transplant therapy, such as transfusion-dependent thalassaemia, then no transplant at all might be the best option.

However, discretion and judgement are required before turning down a matched sibling donor in favour of a poorly matched unrelated cord blood donor, and transplant survival must clearly be a priority of transplant.

## The Future of Cord Blood Transplant in Children

In malignant disease then transplant reduces relapse but its utility in both the malignant and the non-malignant setting is impacted by increased treatment-related toxicity. Strategies for cord transplant therefore and its maintained use as a donor cell source in HCT might address both these aspects.

Most failure in NMD is medicated by graft failure. Engraftment is accelerated by *ex vivo* CD34 stem cell expansion technology and such technologies will likely reduce the risk of graft failure and reduce therefore the risk of cord transplant. Such expansion technologies have been mainly trialed however in malignant diseases. Any expansion must allow preservation of graft T-cells, to permit the graft versus leukaemia effect (Horwitz et al., 2014; Lindemans et al., 2014; Cohen et al., 2020; Horwitz et al., 2021).

We have noticed unprecedented T-cell expansion when granulocytes are given during the transplant. This expansion is transient, and the cells are memory, activated and cytotoxic, and are predominantly CD8 T-cells. Cells of this phenotype mediate the graft versus tumour effect in xenograft studies of cord blood T-cells, and we are testing the use of granulocytes to stimulate such T-cell expansion after cord blood transplant, since immune reconstitution is predominantly otherwise CD4-biased. Certainly, understanding the nature of cord blood graft versus leukaemia response is critical since its refinement may allow the toxicity of mismatched transplant to be reduced, and its efficacy retained (Hiwarkar et al., 2020; Horwitz et al., 2021).

There is clearly a centre effect in cord blood transplant success. There is a reticence from many transplant centres to undertake such transplants, even if their utility in preventing relapse is recognised. We have noticed such in the United Kingdom but protocols for such transplants, and multi-disciplinary team meetings to support the relatively inexperienced centres, have led to increased cord blood uptake. We recommend therefore that such protocols become more widely adopted and that the utility of this donor cell source is preserved and even expanded, as confidence in its use grows.

Cord blood banks need to be maintained and supported, and the number of adequately typed units of good quality increased. Such units will both reduce the time to transplant (as they are typed) and transplant morbidity and mortality (as they are of good quality and HLA match).

We consider that cord blood transplant has a very significant place in paediatric transplantation, and that the change from cord to haploidentical donors is likely in many clinical circumstances to be associated with adverse outcomes for children.
